# Comprehensive analysis of the characteristics and treatment outcomes of patients with non-small cell lung cancer treated with anti-PD-1 therapy in real-world practice

**DOI:** 10.1007/s00432-019-02899-y

**Published:** 2019-03-25

**Authors:** Beung-Chul Ahn, Kyoung-Ho Pyo, Chun-Feng Xin, Dongmin Jung, Hyo Sup Shim, Chang Young Lee, Seong Yong Park, Hong In Yoon, Min Hee Hong, Byoung Chul Cho, Hye Ryun Kim

**Affiliations:** 1 Division of Medical Oncology, Department of Internal Medicine, Yonsei Cancer Center, Yonsei University College of Medicine, 50 Yonsei-ro, Seodaemun-gu, Seoul, 120-752 Republic of Korea; 2 Severance Biomedical Science Institute, Yonsei University College of Medicine, Seoul, Republic of Korea; 3 Department of Pathology, Severance Hospital, Yonsei University College of Medicine, Seoul, Republic of Korea; 4 Department of Thoracic and Cardiovascular Surgery, Yonsei University College of Medicine, Seoul, Republic of Korea; 5 Department of Radiation Oncology, Yonsei Cancer Center, Yonsei University College of Medicine, Seoul, Republic of Korea

**Keywords:** Immunotherapy, Non-small cell lung cancer, Real-world setting, PD-1, Survival

## Abstract

**Purpose:**

Immune checkpoint inhibitors (ICI) have shown marked responses in patients with non-small cell lung cancer (NSCLC) in clinical trials. However, because such trials comprise cohorts selected based on specific criteria, it is unclear if their results represent routine clinical practice.

**Methods:**

We examined 155 patients with advanced NSCLC who were administered either nivolumab or pembrolizumab at Yonsei Cancer Center, Korea between March 2014 and January 2019. Patient characteristics, *EGFR*/*ALK* mutation status, metastatic locations, response to ICIs, and adverse events were retrospectively analyzed.

**Results:**

The median age was 64 years and 72.9% of patients were male; former or current smokers constituted 67.1% of the subjects. Adenocarcinoma was predominant (67.7%), and 50.3% of the patients underwent ≥ 2 previous treatments. Twenty-three patients (14.8%) were *EGFR* mutation- or *ALK* rearrangement-positive. The objective response rate (ORR) was 23.9% [95% confidence interval (CI) 17.4–31.4%]; the median progression-free survival (PFS) and overall survival (OS) were 3.06 (95% CI 1.893–4.21) and 10.25 (95% CI 5.39–15.11) months, respectively. Multivariate analysis identified ECOG performance status, *EGFR* mutation/*ALK* rearrangement status, liver metastasis and PD-L1 proportion as independent predictors of OS. Furthermore, 61.9% of the patients had adverse events of any grade; 38.1% had immune-related adverse events that were associated with PFS and OS on multivariate analysis.

**Conclusions:**

The real-world ORR, PFS, OS, and adverse event profiles were comparable to previous clinical trials despite the patients’ different baseline characteristics. Our findings can aid in establishing effective immunotherapeutic management of NSCLC in routine clinical practice.

**Electronic supplementary material:**

The online version of this article (10.1007/s00432-019-02899-y) contains supplementary material, which is available to authorized users.

## Introduction

Lung cancer is a major cause of cancer-related deaths worldwide (Siegel et al. 2017). Non-small cell lung cancer (NSCLC) accounts for approximately 85% of all lung cancers, of which approximately 70% have non-squamous histologies (Herbst et al. 2008; Travis et al. 1995). In 2016, a total of 24,267 new cases of lung cancer were reported in Korea and 17,399 individuals died of this disease, making it the leading cause of cancer-related death in both sexes (Mortality table of Korea 2015).

Patients with advanced lung cancer eventually develop chemotherapy-resistant disease after treatment with conventional cytotoxic agents, demonstrating the necessity for devising other treatment options for refractory NSCLC. More recently, programmed cell death protein 1 (PD-1)/programmed death-ligand 1 (PD-L1) immune checkpoint inhibitors (ICIs) showed potent activity against metastatic NSCLC in subsets of clinical trials (Borghaei et al. 2015; Brahmer et al. 2015; Herbst et al. 2016). Some randomized phase III trials have reported a statistically significant achievement in overall survival (OS) with ICIs over docetaxel in patients with platinum-refractory NSCLC: the CheckMate 017 and CheckMate 057 trials in patients with squamous and non-squamous NSCLC, respectively (both tested nivolumab, a monoclonal-antibody of PD-1) (Borghaei et al. 2015; Brahmer et al. 2015); the Keynote 010 phase II/III trial with pembrolizumab, which also interacts to PD-1 (participation was restricted to at least 1% PD-L1 expression level on tumor cells) (Herbst et al. 2016). To date, the phase III Keynote 024 and Keynote 042 trials reported that pembrolizumab significantly improves the progression-free survival (PFS) and OS over standard first-line platinum-based chemotherapy; these trials comprise patients with at least 50% PD-L1 and 1% PD-L1 expression in their tumor cells, respectively (Lopes et al. 2018; Reck et al. 2016). Based on all these trials, PD-1 inhibitors including nivolumab and pembrolizumab are now approved as standard anticancer treatments for patients with advanced NSCLC.

However, clinical trials have strict and complex enrollment criteria (Garcia et al. 2017). The line of therapy for patient eligibility is usually pre-determined in trials comparing the efficacy of novel investigational products to previous chemotherapies. Thus, the outcomes of these trials do not necessarily represent real-world patients. In particular, there is limited evidence regarding the real-world efficacy of immunotherapy and related clinical findings in patients who are unsuitable for clinical trials or else are categorized into specific subgroups, including those with poor performance status (PS), those with *EGFR* mutations/*ALK* rearrangements, and those of Asian ethnicity (Borghaei et al. 2015; Brahmer et al. 2015).

Therefore, we conducted this retrospective analysis of 155 unselected patients with advanced NSCLC; to our knowledge, this study is the largest of its kind performed in Korea. We posited that identifying the clinical characteristics that influence the efficacy of immunotherapy in a real-world setting would be beneficial for validating previous observations and devising an effective immunotherapy strategy in routine clinical practice.

## Methods

### Patients and samples

In total, 155 patients with advanced NSCLC who were administered a PD-1 inhibitor (nivolumab or pembrolizumab) at Yonsei Cancer center between March 2014 and January 2019 were enrolled. Clinical data including patient characteristics, driver gene mutation status, metastatic locations, response to immunotherapy, and adverse events were retrospectively collected and analyzed. The patients were treated with nivolumab at a dose of 3.0 mg/kg body weight every 2 weeks or pembrolizumab at 200 mg fixed dose every 3 weeks (which represent the licensed dose and administration method in Korea). This study was approved by the institutional review board (IRB no. 4-2016-0678); the requirement for informed consent was waived.

### Assessments

Patients were assessed for treatment response by computed tomography (CT). Chest CT and abdominal CT were taken every two or three cycles during the treatment. Besides regular follow-up, additional images were acquired according to the physician’s discretion. The clinical response to anti-PD-1 treatment was evaluated using these images according to the Response Evaluation Criteria in Solid Tumors (RECIST), version 1.1 (Eisenhauer et al. 2009). The tumor responses to anti-PD-1 treatment were defined as follows: complete response (CR; the disappearance of all target lesions), partial response (PR; 30.0% or more reduction in the sum of the diameters of the target lesions), progressive disease (PD; 20.0% or more increase in the sum of the diameters of the target lesions), and stable disease (SD; not in category to qualify as PR or PD). To consider the difference between RECIST criteria and immune modified-RECIST the patients who were assessed as PD had reassessment after 4–8 weeks to confirm it. The objective response rate (ORR) was defined as the proportion of patients with CR or PR, while the disease control rate (DCR) was defined as the proportion of patients with CR, PR, or SD. PFS was defined as the time from the start of anti-PD-1 treatment to disease progression or death. OS was defined as time from the start of anti-PD-1 treatment to death by any cause. Adverse events related to anti-PD-1 treatment were stated according to the Common Terminology Criteria for Adverse Events, version 4.0 [Common Terminology Criteria for Adverse Events (CTCAE) v4.0 (2018)].

### Statistical analysis

The Kaplan–Meier method was used to estimate OS and PFS; subgroups were compared using the log-rank test for total number of patients. Additional propensity score matching analysis for each clinical characteristic were done to reduce the bias due to confounding variables. Univariable and multivariable Cox proportional hazard regression models were adopted to determine hazard ratios with 95% confidence intervals (CIs). Multivariate analysis was performed with adjustment for age, sex, smoking status, number of prior treatment lines, mutational status, brain metastasis, liver metastasis, and PD-L1 expression level. Additionally, due to the time-dependent nature of immune-related adverse events (irAEs), we performed 6-week landmark analyses including patients who achieved disease control (for PFS; *n* = 111) and those who were alive (for OS; *n* = 133) at 6 weeks to determine the association between irAEs and survival outcomes. Statistical analyses were performed using SPSS version 23 (IBM Software, Armonk, NY, USA) and GraphPad Prism version 5.00 (GraphPad Software, San Diego, CA, USA).

### Identification of PD-L1 expression

In most cases, tumor PD-L1 expression was determined immunohistochemically using the PD-L1 22C3 pharmDx antibody (Dako North America Inc., Carpinteria, CA, USA) or Ventana PD-L1 SP263 antibody (Ventana Medical Systems, Tucson, AZ, USA) as companion diagnosis. PD-L1 expression levels in tumor cells were determined by the percentage of stained tumor cells in each section, which was estimated in increments of 5% except for a 1% value. Patients with at least 1% of the tumor cells who were stained for PD-L1 were considered positive.

## Results

### Clinicopathologic characteristics of the study population

In total, 155 patients with advanced NSCLC were enrolled (Table 1); the majorities were male (72.9) and aged ≥ 60 years (68.4%). Most patients had adenocarcinoma (67.7%) or squamous carcinoma (30.3%). Twenty-three patients (14.8%) had *EGFR* mutations (*n* = 22) or *ALK* rearrangement (*n* = 1), and 99 (63.9%) were identified as PD-L1 positive. Thirty-four patients (21.9%) had an ECOG performance status score of 2 or higher at the beginning of the treatment. As opposed to clinical trials, the lines of administered therapies were diverse, as 16 (10.3%), 61 (39.4%), and 78 (50.3%) of the patients received anti-PD-1 treatment as first-line, second-line, or subsequent to second-line therapy, respectively. At presentation immediately before immunotherapy, the most frequent site of metastasis was the ipsilateral or contralateral lung (71.0%), followed by the brain (39.4%), bone (32.9%), and adrenal gland (18.1%). By the time of data lock (January 30, 2019), 49 patients (31.6%) were alive, 92 (59.4%) had died, and 14 (9.0%) was lost to follow-up. The median follow-up duration for the patients was 17.0 months.

**Table 1:** Baseline characteristics

	*n* (%)
Age (years)	
Median (range)	64 (35–85)
< 60	49 (31.6)
Sex	
Male	113 (72.9)
Female	42 (27.1)
Histology	
Adenocarcinoma	105 (67.7)
Squamous	47 (30.3)
Pleomorphic	2 (1.3)
Unknown	1 (0.6)
Smoking	
Never	51 (32.9)
Former smoker	61 (39.4)
Current smoker	43 (27.7)
*EGFR* and *ALK*	
Wild-type (both)	131 (84.5)
Mutant	23 (14.8) (*ALK*: *n* = 1)
Unknown	1 (0.7)
ECOG PS score	
0	23 (14.8)
1	98 (63.2)
2	20 (12.9)
3	14 (9.0)
Prior treatment lines	
0	16 (10.3)
1	61 (39.4)
2	32 (20.6)
3	28 (18.1)
4	8 (5.2)
≥ 5	10 (6.4)
Metastasis present	
Lung ipsilateral	95 (61.3)
Lung contralateral	79 (51.0)
Brain	61 (39.4)
Bone	51 (32.9)
Adrenal gland	28 (18.1)
Liver	24 (15.5)

*ECOG PS* Eastern Cooperative Oncology Group performance status, *EGFR* epidermal growth factor receptor, *ALK* anaplastic lymphoma kinase

### Treatment outcomes and potential predictors

**Fig. 1:**
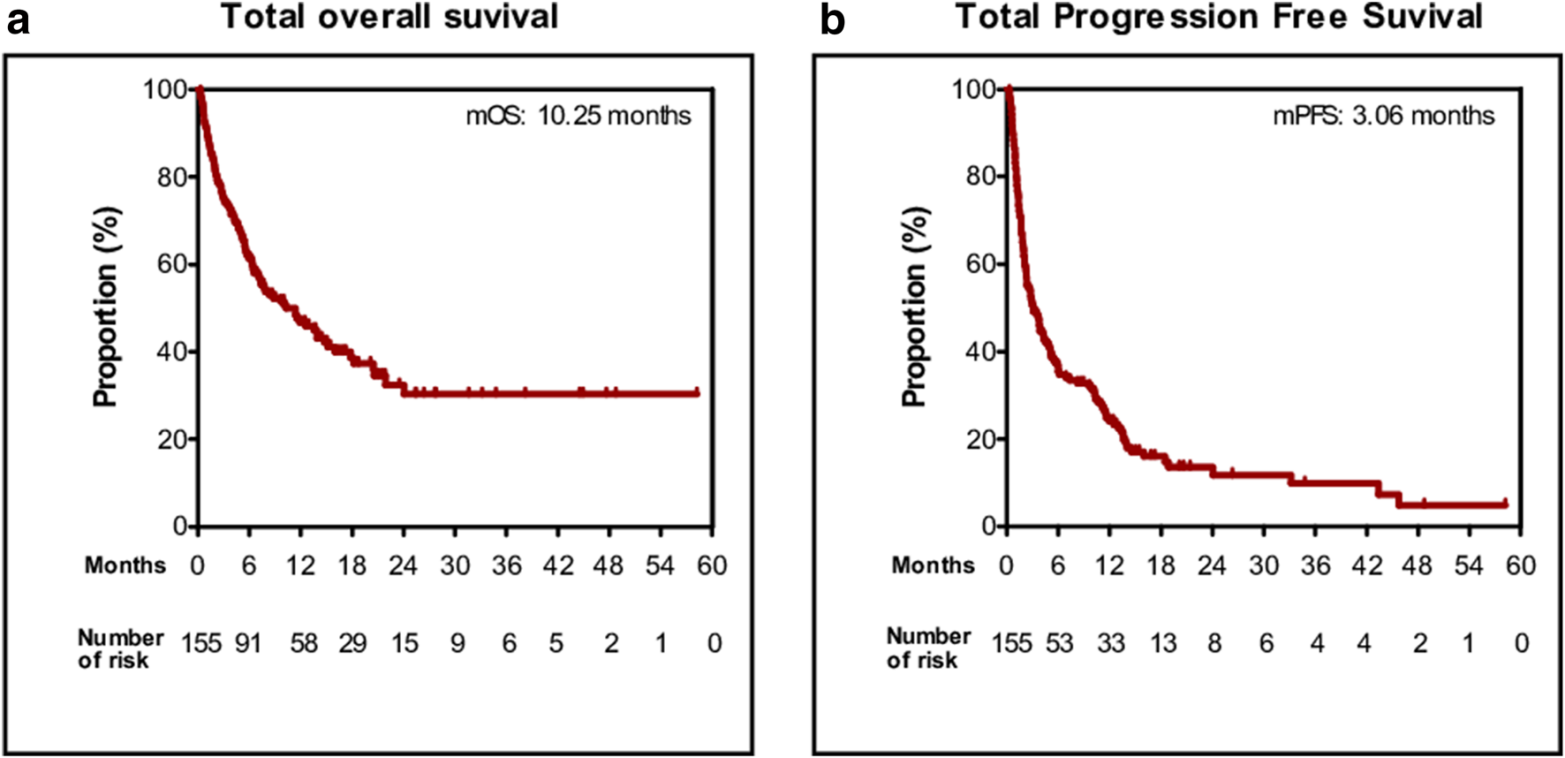
Kaplan–Meier plot for the total population (*n* = 139). **a** Overall survival (OS) and **b** progression-free survival (PFS) from the beginning of anti-PD-1 treatment. *PD-L1* programmed death-ligand 1

The treatment outcomes for all patients are shown in S1 Table. The ORR was 23.9% (*n* = 37), all based on achieving PR. Moreover, 35.5% of the patients had SD, 29.7% had PD, and 11.0% were not evaluated. The median OS and PFS were 10.25 months (95% CI 5.39–15.11) and 3.06 months (95% CI 1.89–4.21), respectively, for all patients (Fig. 1).

We further investigated patients stratified by clinicopathologic factors. ORRs of patients with PS scores of 0–1 and 2–4 were 29.8% and 2.9%, respectively; the DCRs of these subgroups were 71.9% and 14.7%, respectively. The ORRs of patients who were never-smokers, were *EGFR* mutation/*ALK*-rearrangement-positive, and had ≥ 50% PD-L1 expression were 13.7%, 13.0%, and 37.7%, respectively; the corresponding DCRs were 51.0% and 34.7%, and 75.4%, respectively.

**Fig. 2:**
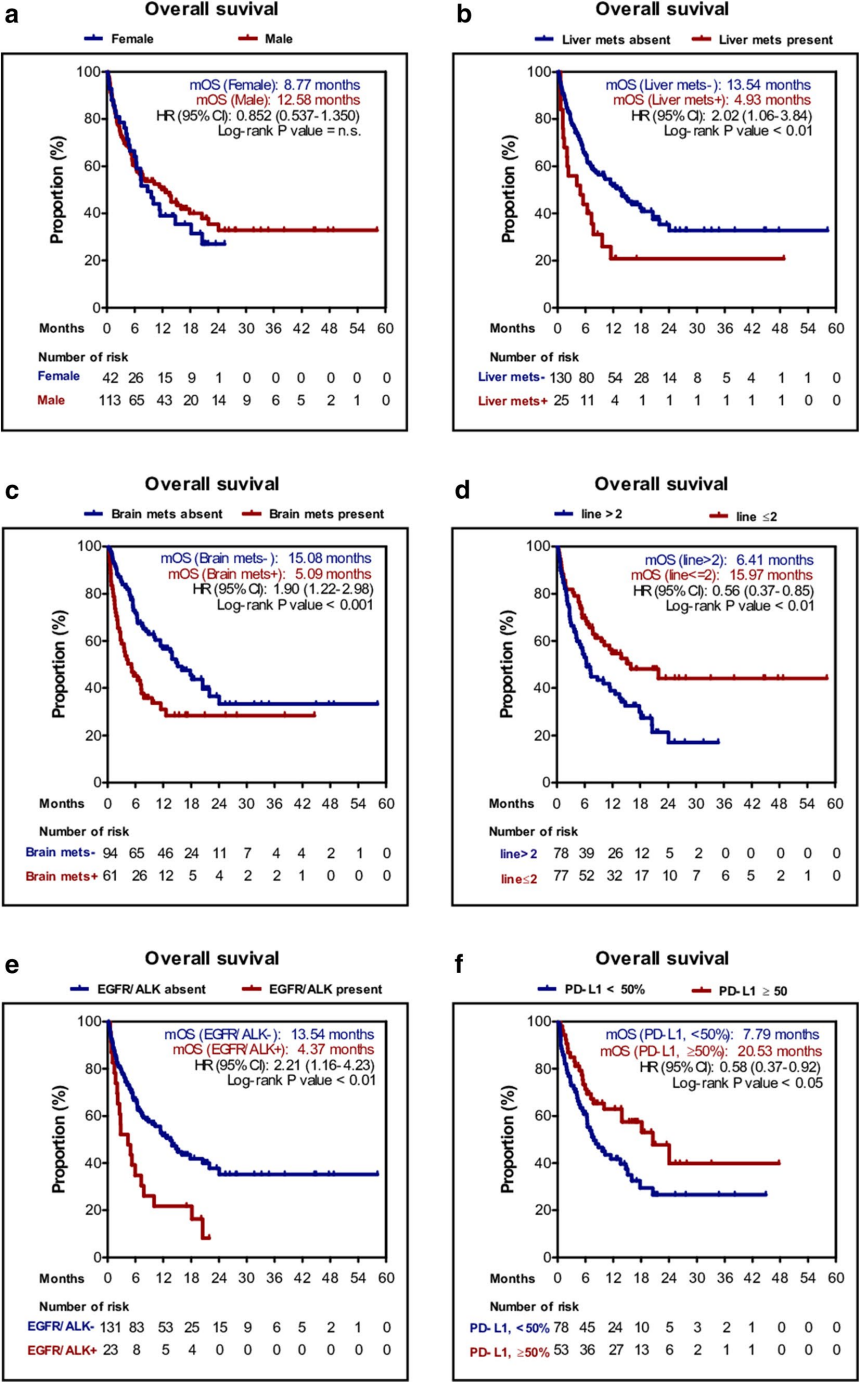
Kaplan–Meier plot for the overall survival (OS) stratified by clinical factors. **a** Sex; **b** liver metastasis; **c** brain metastasis; **d** line of therapy; **e** EGFR/ALK mutation status; **f** PD-L1 expression level of 50%. *PD-L1* programmed death-ligand 1, *EGFR* epidermal growth factor receptor, *ALK* anaplastic lymphoma kinase, *HR* hazard ratio, *n.s*. not significant, *UD* undetermined

We next performed PFS and OS analyses of patients stratified by the above factors and metastatic lesions. Age, sex, and smoking status did not significantly influence OS and PFS. However, median PFS rates of patients with *EGFR* mutation/*ALK* rearrangement were significantly shorter than those of wild-type patients (1.6 vs. 3.8 months; *P* < 0.01) as were OS rates (4.4 vs. 13.5 months; *P* < 0.01). Furthermore, the median PFS of patients with 50% or more PD-L1-positive cells was 6.0 months, which was significantly longer than those with 0–49% PD-L1-positive cells (2.9 months; *P* < 0.01); the same was true for OS (20.5 vs. 7.8 months, *P* = 0.021). Other factors that showed significant differences were PS score, the presence of metastatic lesions (i.e., in the brain and liver), and line of therapy. Kaplan–Meier plots are shown in Fig. 2 and S1 Fig.

Multivariate analysis identified poor PS, *EGFR* mutation/*ALK* rearrangement positivity, liver metastasis and low PD-L1 expression as independent negative predictors of OS (Table 2).

**Table 2:** Cox proportional hazards regression analysis of the effects of clinical factors on overall survival

Category	Univariate			Multivariate		
	HR	95% CI	*P* value	HR	95% CI	*P* value
Age (< 75 vs. ≥ 75 years)	1.049	0.593–1.854	0.871	0.712	0.337–1.502	0.372
Sex (male vs. female)	1.167	0.748–1.822	0.496	0.527	0.150–1.848	0.317
ECOG PS (0– 1 vs. 2– 3)	6.989	4.391–11.12	< 0.001	7.566	4.008–14.282	< 0.001
Smoking (never vs. current or former)	1.022	0.645–1.620	0.925	0.878	0.258–2.987	0.835
PD-L1 (< 50% vs. ≥50%)	0.631	0.398–0.999	0.049	0.430	0.250–0.741	0.002
Liver metastases (absent vs. present)	2.045	1.228–3.406	0.006	2.388	1.263–4.513	0.007
Brain metastases (absent vs. present)	1.926	1.272–2.917	0.002	1.601	0.935–2.741	0.086
Prior treatment line (≥ 2 vs. <2)	1.777	1.170–2.700	0.007	1.202	0.685–2.109	0.521
*EGFR* mutation or *ALK* rearrangement (absent vs. present)	2.230	1.352–3.676	0.002	2.711	1.377–5.338	0.0024

*HR* hazard ratio, *CI* confidence interval, *ECOG PS* Eastern Cooperative Oncology Group performance status (score), *PD-L1* programmed death-ligand 1

### Brain and liver metastasis response

We also analyzed the treatment outcomes of patients according to brain and/or liver metastasis status. Sixty-one patients had brain metastasis upon commencing ICI therapy, 41 of whom underwent local radiotherapy before or during treatment. Response in the brain was evaluated via magnetic resonance imaging during the same global response evaluation cycle. The patients’ intracranial ORR and DCR were 16.4% (95% CI 8.16–28.10%) and 42.6% (95% CI 30.02–55.92%), respectively. The ORR did not differ significantly from the global response rate (16.4% vs. 23.9%, *P* = 0.23), but the DCRs were significantly different (42.6% vs. 59.4%, *P* = 0.026). Furthermore, 25 patients had liver metastasis; their ORR and DCR were 12.0% (95% CI 2.55–31.22%) and 32.0% (95% CI 14.95–53.50%), respectively, and only the DCR differed significantly from the global response (ORR, *P* = 0.19; DCR, *P* = 0.011). The treatment responses of each region for patients with brain and liver metastases are shown in S2 Table.

### Adverse events and their association with clinical outcomes

The proportion of patients who experienced adverse events of any type and grade was 61.9%. The three most common adverse events were rash (19.4%), decreased appetite (12.3%), and fatigue (11.6%); none were grade ≥ 3. The most common grade ≥ 3 adverse event was pneumonia (6.5%) followed by pneumonitis (3.2%).

Based on the previous studies, we defined irAEs as adverse events with a potential immunological basis that require immunosuppressive or endocrine therapy (Friedman et al. 2016). We strictly considered only those irAEs that medical professionals could recognize objectively via physical examination or laboratory results; this also helped to reduce bias. IrAEs observed in our patients are shown in Table 3. The pneumonitis was distinguished from pneumonia by sputum culture plus laboratory C-reactive protein (CRP) and procalcitonin level. Most of patients who were diagnosed as pneumonitis had process of multidisciplinary team meeting and/or consultation to pulmonologists (S3 Table).

**Table 3:** Treatment-related adverse events according to category and grade

Adverse events	No. of subjects (all grades)	Percentage (all grades)	No. of subjects (grades 3–4)	Percentage (grades 3–4)
Any AEs				
Decrease appetite	19	12.3	0	0
Fatigue	18	11.6	0	0
Dyspnea	16	10.3	1	0.7
Pneumonia	15	10.3	10	6.5
Nausea/vomiting	4	2.6	0	0
Pyrexia	3	1.9	0	0
Constipation	3	1.9	0	0
Edema	2	1.3	0	0
Neuropathy	1	0.7	0	0
Infusion reaction	1	0.7	0	0
Immune-related AEs				
Rash	30	19.4	0	0
Pneumonitis	11	12.2	5	3.2
Diarrhea	10	6.5	1	0.7
Hypothyroidism	10	6.5	0	0
AST/ALT elevation	5	3.2	0	0
Hemolytic anemia	3	1.9	1	0.7
Adrenal insufficiency	3	1.9	0	0
Panhypopituitarism	1	0.7	1	0.7

*AE* adverse event, *ALT* alanine aminotransferase, *AST* aspartate aminotransferase

**Fig. 3:**
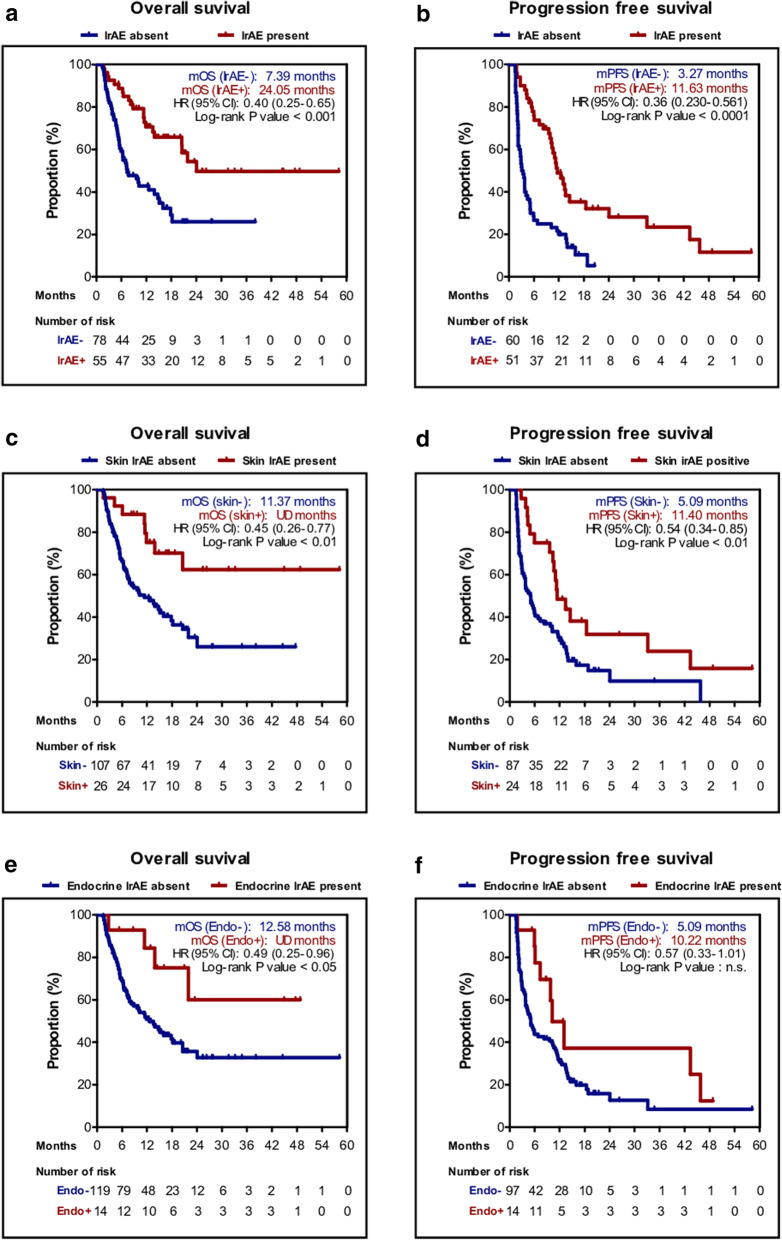
Kaplan–Meier plot with 6-week landmark analysis for the overall survival (OS) and progression-free survival (PFS) stratified by the presence of irAEs. By any irAEs **a** OS, **b** PFS; by skin irAEs **c** OS, **d** PFS; by endocrine irAEs **e** OS, **f** PFS. *irAE* immune-related adverse event, *HR* hazard ratio, *n.s*. not significant, *UD* undetermined

We categorized hypothyroidism, adrenal insufficiency, and panhypopituitarism as endocrine irAEs for further analysis. The proportion of patients who experienced irAEs was 38.1% and 5.16% experienced grade ≥ 3 irAEs. Six-week landmark analysis showed that the ORR of patients with irAEs was higher than in those without, although not significantly different [21 of 51 patients (41.2%) vs. 16 of 60 patients (26.7%); *P* = 0.11]. However, the development of irAEs was significantly associated with longer PFS [11.63 months (95% CI 9.21–14.05) vs. 3.27 months (95% CI 2.01–4.17); *P* < 0.001] and OS [24.05 months (95% CI NR–NR) vs. 7.39 months (95% CI 3.49–11.29); *P* < 0.001]. Upon further analysis of irAE subtypes, patients with skin rash had significantly longer PFS [11.40 months (95% CI 7.87–14.93) vs. 5.09 months (95% CI 3.53–6.66); *P* = 0.008] and OS [NR (95% CI NR–NR) vs. 11.37 months (95% CI 5.84–16.89); *P* = 0.004]. Patients with endocrine irAEs had longer PFS [10.22 months (95% CI 6.04–14.39) vs. 5.09 months (95% CI 3.49–6.70); *P* = 0.054] and OS [NR (95% CI NR–NR) vs. 12.58 months (95% CI 8.02–17.15); *P* = 0.037], although the differences were not significant for PFS (Fig. 3).

Multivariate analysis revealed that the presence of any irAEs was significantly associated with increased PFS and OS based on the 6-week landmark analysis, whereas skin irAEs and endocrine irAEs subsets were not. Overall, skin and endocrine irAEs were not identified as significant positive predictive factors, though they showed a tendency as such (Table 4).

**Table 4:** Cox proportional hazards regression analysis of the effect of irAE development on PFS and OS (6-week landmark)

Survival	Univariate			Multivariate		
	HR	95% CI	*P* value	HR	95% CI	*P* value
PFS (6-week landmark), *N* = 111						
Any irAEs	0.367	0.233–0.579	< 0.001	0.434	0.256–0.735	0.002
Skin irAEs	0.488	0.284–0.837	0.009	0.643	0.350–1.180	0.154
Endocrine irAEs	0.503	0.246–1.027	0.059	0.368	0.132–1.028	0.057
Pneumonitis irAE	0.685	0.276–1.702	0.416	1.686	0.618–4.597	0.307
OS (6-week landmark), *N* = 133						
Any irAEs	0.383	0.228–0.644	< 0.001	0.484	0.255–0.919	0.027
Skin irAEs	0.350	0.167–0.734	0.005	0.420	0.162–1.087	0.074
Endocrine irAEs	0.358	0.130–0.983	0.046	0.255	0.051–1.288	0.098
Pneumonitis irAE	1.252	0.503–3.113	0.629	4.117	1.420–11.942	0.009

Covariables included age (≥ 75 vs. < 75 years), sex (male vs. female), Eastern Cooperative Oncology Group performance status score (0–1 vs. 2–3), smoking status (current or former vs. never), PD-L1 (< 50% vs ≥ 50%), liver metastasis present (yes vs. no), brain metastasis present (yes vs. no), number of prior treatment lines (≥ 2 vs. < 2), and *EGFR* mutation or *ALK* rearrangement (absent vs. present)

*irAE* immune-related adverse events, *EGFR* epidermal growth factor receptor, *ALK* anaplastic lymphoma kinase, *PFS* progression-free survival, *OS* overall survival, *HR* hazard ratio, *CI* confidence interval

Additional 12-week and 24-week landmark analysis were also done and are shown on online resource (S4 Table and S2 Fig.) The result of these analyses showed similar trend as 6-week landmark but 12-week analysis only showed significance between the presence of any irAEs and OS not with PFS. 24-week analysis did not show significance and only showed similar trends.

## Discussion

Currently, the National Clinical Cancer Network and American Society of Clinical Oncology guidelines do not recommend more than two consecutive lines of cytotoxic chemotherapy for advanced NSCLC; intervening or subsequent immunotherapy, best supportive care, or participation in a clinical trial is otherwise recommended (Rizvi et al. 2015; Rozenblum et al. 2017). In contrast to chemotherapy or tyrosine kinase inhibitors, ICIs have the ability to restore a patient’s antitumor immunity, allowing the destruction of malignant cells with the potential for a robust and durable clinical response (Wang et al. 2014). In the CheckMate 017 (squamous NSCLC) and CheckMate 057 (non-squamous NSCLC) clinical trials for nivolumab, the ORRs were 20.0% and 19.0%, median PFS rates were 3.5 and 2.3 months, and median OS rates were 9.2 and 12.2 months, respectively (Borghaei et al. 2015; Brahmer et al. 2015). The Keynote 001 study of pembrolizumab for patients with advanced NSCLC reported an objective response rate of 19.4%, median PFS of 3.7 months, and median OS of 12 months (Garon et al. 2015). Hence, immunotherapy has become an indispensable option for any line of therapy for advanced NSCLC.

The ORR, PFS, and OS in our study (23.9%, 3.06 months, and 10.25 months, respectively) were comparable to those of the previous studies. Although we enrolled unselected patients who were ineligible for the previous trials or participated as minor subset cohorts (including those with poor PS, *EGFR* mutations/*ALK* rearrangements, and brain and liver metastases, which are factors known to be indicators of unfavorable responses to immunotherapy) (Tamiya et al. 2018), the clinical outcomes were similar to the previously published results. This could be partially explained by higher PD-L1 expression (40.5% of patients in our study had PD-L1 expression levels ≥ 50%; whereas 23.3% of patients exhibited PD-L1 levels ≥ 50% in the Keynote-001 study and 37.2% exhibited PD-L1 levels ≥ 10% in the CheckMate-057 study) (Borghaei et al. 2015; Garon et al. 2015). Higher PD-L1 expression is reportedly a favorable predictor of immunotherapy efficacy, and levels of this protein are usually higher among Asians (Lin et al. 2016; Patel and Kurzrock 2015). Furthermore, ethnicity itself could have been a factor, since the abovementioned studies comprised mostly Caucasian patients, whereas ours were Asian.

Our data indicated that harboring *EGFR* mutations/*ALK* rearrangements was associated with poorer PFS and OS, which was consistent with recent retrospective and prospective studies (Lee et al. 2017). Preliminary studies suggest that sensitivity to ICIs is high in tumors with high somatic mutations (Rizvi et al. 2015; Rozenblum et al. 2017). Therefore, never-smokers and patients with *EGFR* mutations/*ALK* rearrangements are known to have poorer outcomes because of their low mutational burdens, although our study did not reveal an association between smoking status and survival outcomes. Subgroup analyses of both the CheckMate 057 (Borghaei et al. 2015) and Keynote 010 (Herbst et al. 2016) prospective trials revealed no significant OS differences based on *EGFR* mutation status. Overall, excluding patients with *EGFR* mutations/*ALK* rearrangements ought to produce a higher response rate to anti-PD-1 immunotherapy in routine clinical practice.

Our results also suggested that PD-L1 expression levels ≥ 50% were well-correlated with improved PFS and OS rates following anti-PD-1 treatment. As PD-L1 expression alone is accepted as an imperfect biomarker for predicting prognosis, it remains debated whether PD-L1 expression levels should be used as a benchmark for prescribing ICIs (Herbst et al. 2014; Patel and Kurzrock 2015). However, our results imply that determining PD-L1 status can help identify patients most likely to benefit from anti-PD-1 treatment in real-world settings.

The existence of liver or brain metastases at the commencement of immunotherapy was associated with poor PFS and OS in our study. Patients with advanced NSCLC who have liver or brain metastases and are receiving chemotherapy or tyrosine kinase inhibitors are known to have poorer prognoses than those with metastases in other locations (Hoang et al. 2012). There are only few retrospective studies regarding immunotherapy outcomes and their association with metastatic lesions. One such retrospective study showed a relationship between the metastatic site and PFS in patients with advanced NSCLC who were treated with nivolumab (Tamiya et al. 2018), while another found that nivolumab was effective against brain metastasis (Gauvain et al. 2018). In our study, we revealed not only differences in ORR and PFS, but also in OS in patients who received anti-PD-1. This supports considering the metastatic lesion site a criterion for selecting candidates for immunotherapy.

Finally, our data indicated that the development of certain irAEs is associated with improved anti-PD-1 treatment efficacy in patients with NSCLC, which is consistent with earlier studies (Suresh et al. 2018; Teraoka et al. 2017). A previous retrospective study showed that thyroid dysfunction irAE is associated with a better prognosis in patients with NSCLC (Osorio et al. 2017). Several other retrospective studies have demonstrated similar associations in patients with dermatological irAEs who were treated for melanoma and NSCLC with ICIs (Freeman-Keller et al. 2016; Hasan Ali et al. 2016). Most recently, Haratani et al. performed a landmark analysis that revealed significant differences in both PFS and OS between NSCLC patients with vs. without irAEs [hazard ratios of 0.525 (95% CI 0.287–0.937); *P* = 0.03 for PFS and 0.282 (95% CI 0.101–0.667); *P* = 0.003 for OS on multivariate analysis] (Haratani et al. 2018; Teraoka et al. 2017) Our own 6-week landmark analysis revealed significant differences in OS and PFS when patients were stratified by existence of irAEs, also multivariate analysis confirmed a significant difference for PFS and OS. Our study included a greater number of covariables than those examined by Haratan et al.; we additionally investigated PD-L1 status, PS, and liver metastasis (Table 4). Our comprehensive landmark analyses suggest that the early onset of irAEs is predictive of response or of the durable clinical benefits in patients with NSCLC treated with PD-1 inhibitors, thereby possibly aiding clinicians in improving immunotherapy planning, including whether to switch or cease treatment, during the interval before the routine response evaluation.

To the best of our knowledge, ours is one of the largest comprehensive retrospective studies of real-world patients who were treated with PD-1 inhibitors. Previous retrospective studies examined real-world situations only partially, and their findings were, therefore, confined in scope. For example, some studies included only patients who were previously treated, while others only considered patients who received a single immunotherapy agent. Furthermore, due to insufficient follow-up periods, most of the previous retrospective studies were unable to determine matured OS dates (Fujimoto et al. 2018; Garassino et al. 2018; Kobayashi et al. 2018). In contrast, our study comprised near-complete results for both efficacy and safety; in addition, it compared the outcomes of patients with specific clinical factors and adverse events in detail.

In conclusion, our study provided comprehensive clinical characteristics and outcomes of patients with NSCLC who received anti-PD-1 treatment in Korea. In the context of heterogeneous real-world settings, further efforts are required to develop efficient therapeutic strategies, ranging from proper patient selection to determining the correct timing of administering immunotherapy.

## Electronic supplementary material

Below is the link to the electronic supplementary material.

Supplementary material 1 (DOCX 30 KB)

Supplementary material 2 (TIFF 13029 KB)

Supplementary material 3 (TIFF 4442 KB)
