# Supplementary material for: Comprehensive analysis of the characteristics and treatment outcomes of patients with non-small cell lung cancer treated with anti-PD-1 therapy in real-world practice
Source: J Cancer Res Clin Oncol. 2019 Mar 25;145(6):1613–23. doi: 10.1007/s00432-019-02899-y (PMC6527531; doi:10.1007/s00432-019-02899-y)
Supplement: Supplementary file 1 — Supplementary material 1 (DOCX 30 KB) [file 432_2019_2899_MOESM1_ESM.docx]

| **Supplemental Table 1. Efficacy outcomes** | |
| --- | --- |
| **Best overall response** | **Total N=155**  **n (%)** |
| Complete response | 0 (0) |
| Partial response | 37 (23.9) |
| Stable disease | 55 (35.5) |
| Progressive disease | 46 (29.7) |
| Not evaluated | 17 (11.0) |
| Objective response rate, n (%) (95% CI) | 37 (23.9) (17.42–31.40) |

Abbreviation: CI = confidence interval.

| **Supplemental Table 2. Efficacy outcomes by site of metastasis (brain and liver)** | |
| --- | --- |
| **Best overall response**  **Brain** | **Total N=61**  **n (%)** |
| Complete response | 1 (1.6) |
| Partial response | 9 (14.8) |
| Stable disease | 16 (26.2) |
| Progressive disease | 12 (19.7) |
| Not evaluated | 23 (37.7) |
| Objective response rate, n (%) (95% CI) | 10 (16.4) (8.16–28.10) |
| **Best overall response**  **Liver** | **Total N=25**  **n (%)** |
| Complete response | 1 (4.0) |
| Partial response | 2 (8.0) |
| Stable disease | 5 (20.0) |
| Progressive disease | 11 (44.0) |
| Not evaluated | 6 (24.0) |
| Objective response rate, n (%) (95% CI) | 3 (12.0) (2.55–31.22) |

Abbreviation: confidence interval.

**Supplemental Table 3. Clinical process and lab results of patients who were diagnosed as pneumonitis irAE.**

|  | **Sputum Culture** | **CRP**  ***(0~8mg/L)** | **Procalcitonin**  ***(0.00~0.50ng/mL)** | **Pulmonology consult** | **Treatment** | **Follow up** | |
| --- | --- | --- | --- | --- | --- | --- | --- |
| Patient 1 | α-streptococcus  (non pathogen) | 16.9 | 0.08 | Yes | steroid | resolved | |
| Patient 2 | negative | 78.0 | 0.08 | No | steroid | resolved | |
| Patient 3 | α-streptococcus  (non pathogen) | 124.2 | 0.10 | Yes | steroid | resolved | |
| Patient 4 | negative | 91.1 | 0.09 | Yes | steroid | f/u loss | |
| Patient 5 | negative | 107.5 | 0.13 | No | conservative care | f/u loss | |
| Patient 6 | negative | 34.3 | 1.64 | Yes | steroid | resolved | |
| Patient 7 | negative | 62.9 | <0.02 | Yes | steroid | resolved | |
| Patient 8 | negative | 52.5 | 0.2 | Yes | steroid | f/u loss | |
| Patient 9 | negative | 51.1 | NA | Yes | conservative care | expired | |
| Patient 10 | NA | NA | NA | No | steroid | resolved | |
| *CRP, Procalcitonin reference rage  Abbreviation: CRP, C-reactive protein | | | | | | |  |

| **Supplemental Table 4.**  **Cox proportional hazards regression analysis of the effect of irAE development on PFS and OS (12-week landmark)** | | | | | | |
| --- | --- | --- | --- | --- | --- | --- |
| Survival | Univariate | | | Multivariate | | |
| **PFS (12-week landmark)**  **N=77** | **HR** | **95% CI** | **P-value** | **HR** | **95% CI** | **P-value** |
| Any irAEs | 0.519 | 0.297–0.908 | 0.021 | 0.559 | 0.290–1.080 | 0.083 |
| Skin irAEs | 0.707 | 0.389–1.284 | 0.255 | 0.890 | 0.446–1.774 | 0.740 |
| Endocrine irAEs | 0.658 | 0.300–1.443 | 0.296 | 0.523 | 0.172–1.590 | 0.253 |
| Pneumonitis irAE | 0.642 | 0.198–2.084 | 0.460 | 1.928 | 0.450–8.257 | 0.376 |
|  |  |  |  |  |  |  |
| **OS (12-week landmark)**  **N=114** | **HR** | **95% CI** | **P-value** | **HR** | **95% CI** | **P-value** |
| Any irAEs | 0.374 | 0.208–0.672 | 0.001 | 0.442 | 0.223–0.873 | 0.019 |
| Skin irAEs | 0.374 | 0.168–0.833 | 0.016 | 0.453 | 0.172–1.194 | 0.109 |
| Endocrine irAEs | 0.326 | 0.102–1.049 | 0.060 | 0.128 | 0.013–1.298 | 0.082 |
| Pneumonitis irAE | 1.341 | 0.483–3.723 | 0.574 | 3.910 | 1.117–13.684 | 0.033 |

| **Cox proportional hazards regression analysis of the effect of irAE development on PFS and OS (24-week landmark)** | | | | | | |
| --- | --- | --- | --- | --- | --- | --- |
| Survival | Univariate | | | Multivariate | | |
| **PFS (24-week landmark)**  **N=53** | **HR** | **95% CI** | **P-value** | **HR** | **95% CI** | **P-value** |
| Any irAEs | 1.013 | 0.452–2.270 | 0.976 | 1.348 | 0.545–3.332 | 0.518 |
| Skin irAEs | 0.789 | 0.371–1.681 | 0.539 | 1.216 | 0.483–3.057 | 0.678 |
| Endocrine irAEs | 1.122 | 0.453–2.781 | 0.804 | 1.106 | 0.277–4.414 | 0.886 |
| Pneumonitis irAE | 0.375 | 0.050–2.826 | 0.341 | 9.167 | 0.690–121.70 | 0.093 |
|  |  |  |  |  |  |  |
| **OS (24-week landmark)**  **N=91** | **HR** | **95% CI** | **P-value** | **HR** | **95% CI** | **P-value** |
| Any irAEs | 0.374 | 0.208–0.672 | 0.001 | 0.618 | 0.281–1.357 | 0.230 |
| Skin irAEs | 0.374 | 0.168–0.833 | 0.016 | 0.533 | 0.173–1.645 | 0.274 |
| Endocrine irAEs | 0.326 | 0.102–1.049 | 0.060 | 0.454 | 0.056–3.710 | 0.461 |
| Pneumonitis irAE | 1.823 | 0.554–5.997 | 0.323 | 5.115 | 1.110­–23.574 | 0.036 |
