# Supplementary figures and images for: Comprehensive analysis of the characteristics and treatment outcomes of patients with non-small cell lung cancer treated with anti-PD-1 therapy in real-world practice
Source: J Cancer Res Clin Oncol. 2019 Mar 25;145(6):1613–23. doi: 10.1007/s00432-019-02899-y (PMC6527531; doi:10.1007/s00432-019-02899-y)

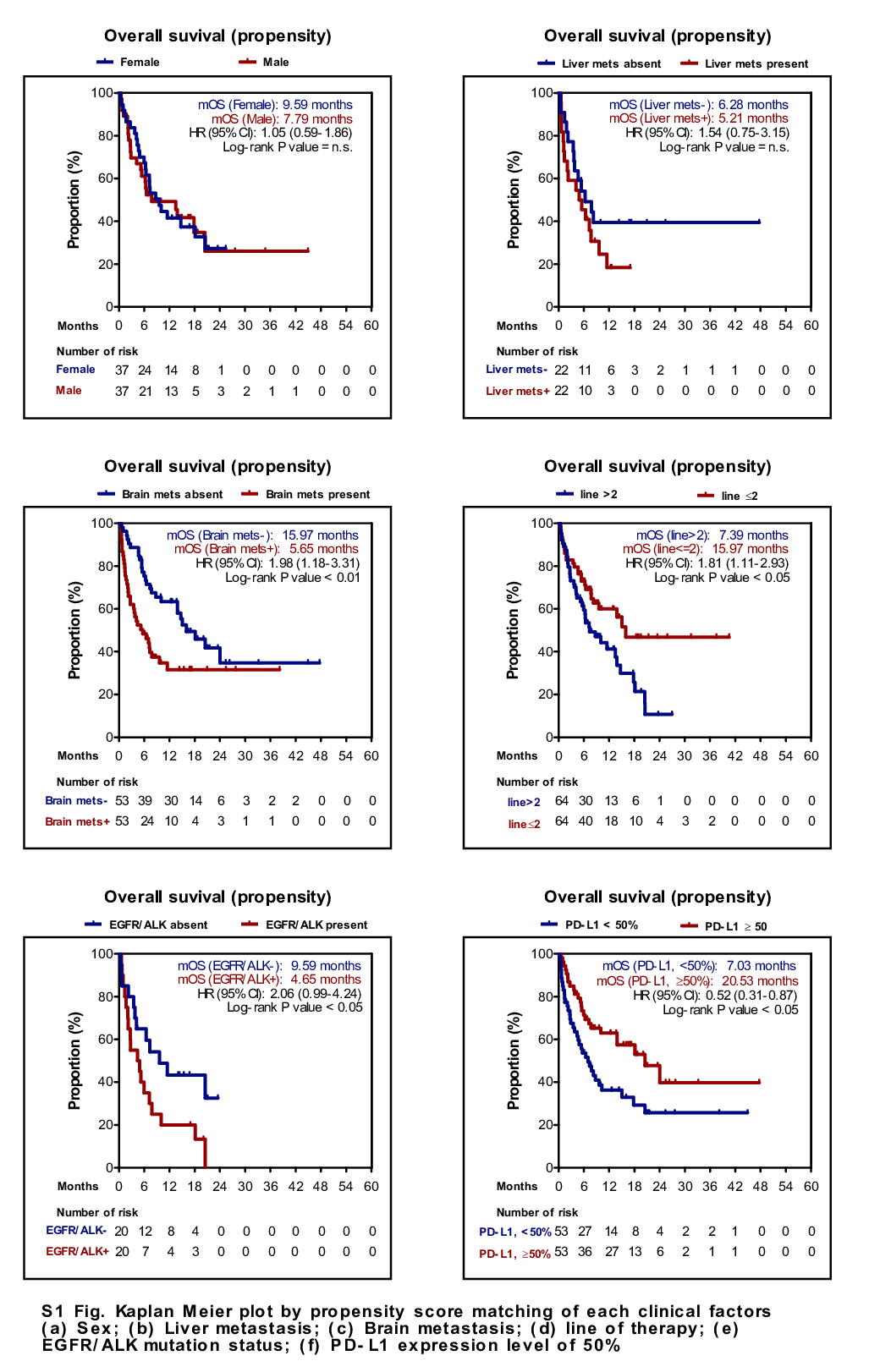

Supplement: Supplementary file 2 — Supplementary material 2 (TIFF 13029 KB) [file 432_2019_2899_MOESM2_ESM.tiff]

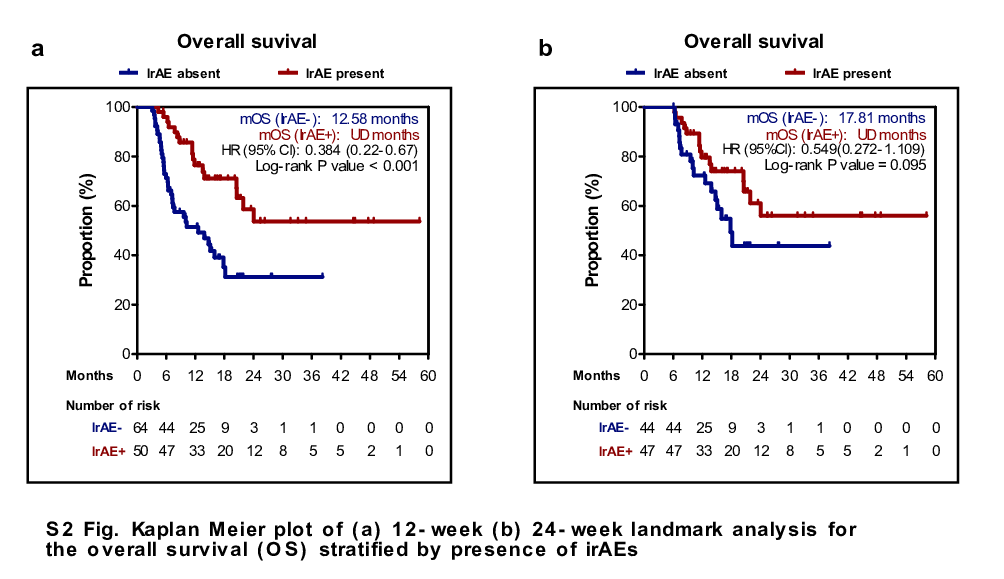

Supplement: Supplementary file 3 — Supplementary material 3 (TIFF 4442 KB) [file 432_2019_2899_MOESM3_ESM.tiff]
